# The mitochondrial genome of *Cofana yasumatsui* (Hemiptera: Cicadellidae: Cicadellinae)

**DOI:** 10.1080/23802359.2020.1721371

**Published:** 2020-02-07

**Authors:** Li-Kun Zhong, Mao-Fa Yang, Xiao-Fei Yu

**Affiliations:** a College of Agriculture, Guizhou University, Guiyang, China;; b Guizhou Provincial Key Laboratory for Agricultural Pest Management of the Mountainous Region, Institute of Entomology, Guizhou University, Guiyang, China;; c College of Tobacco Science, Guizhou University, Guiyang, China;; d Guizhou Key Laboratory of Tobacco Quality Research, Guiyang, Guizhou, China

**Keywords:** Mitochondrial genome, *Cofana yasumatsui*, Cicadellinae, molecular phylogeny

## Abstract

The complete mitochondrial genome of *Cofana yasumatsui* (Hemiptera: Cicadellidae: Cicadellinae) is sequenced. Based on annotation, the mitochondrial genome is 15,019 bp, has an A + T content of 77.2% (A = 42.0%, T = 35.2%, G = 10.0%, C = 12.8%), which is the classical structure for insect mitogenome. All PCGs started with ATN, except *ATP8* and *ND5* starting with TTG. All PCGs used TAN as stop codon, except *ND5* stopping with single T. The phylogenetic relationship of *C. yasumatsui* clustered together with *Cicadella viridis*, *Homalodisca coagulata*, *Homalodisca vitripennis,* and *Bothrogonia ferruginea* from Cicadellinae. This is identical with the result of the traditional taxonomy.

The leafhopper genus *Cofana* was established by Melichar (1926), belonging to the tribe Cicadellini of subfamily Cicadellinae (Hemiptera: Cicadellidae). This genus now records 10 species in China (Yang et al. 2017), all species are very similar in coloration and difficult to distinguish externally. Moreover, most of them had strong phototaxis. The complete mitochondrial genome of *Cofana yasumatsui* (Young, 1979) was firstly conducted by next-generation sequencing method, which will facilitate future research on identification, population genetics, and evolution of the leafhopper subfamily Cicadellinae.

Total genome DNA was extracted from male adult of *C. yasumatsui* which was collected from Gaoligong Mountain, Yunnan Province, China (25°18′20″N, 98°47′42″E) in August 2018. Also, voucher specimen’s genome DNA and male external genitalia are deposited in the Institute of Entomology, Guizhou University, Guiyang, China (GUGC), the accession number is GUGC-IDT-00189. The complete mitogenome of *C. yasumatsui* is 15,019 bp in length (GenBank accession number MN793964), containing 13 protein-coding genes (PCGs), 22 transfer RNA genes (tRNA), 2 ribosomal RNA genes (rRNA), and 1 large noncoding region (C-region). In general, the *C. yasumatsui* mitogenome has an A + T content of 77.2% (A = 42.0%, T = 35.2%, G = 10.0%, C = 12.8%). The AT-skew and GC-skew are positive (0.09) and negative (−0.12). The most PCGs started with ATN (ATA, ATC, ATT, ATG), except *ATP8* and *ND5*, starting with TTG. All PCGs used TAN as stop codon, except *ND5* using incomplete single T as stop codon. Identification of all tRNA genes using ARWEN version 1.2 software (Laslett and Canbäck 2008). The *16S* rRNA gene is 1202 bp in size, located between *tRNA-L2* and *tRNA-V*; the *12S* rRNA gene is 818 bp in length, located after *tRNA-V*. The control region is located between *12S* rRNA and *tRNA-I*.

Phylogenetic relationship reconstruction based on 13 PCGs tandem nucleotide sequences was analyzed by MrBayes (Figure 1). The 13 PCG sequences without stop codons were used in the phylogenetic analysis. Using the MAFFT algorithm in the TranslatorX online server, multiple alignments of each PCG and codon-based were analyzed (Abascal et al. 2010). Phylogenetic trees were generated using MrBayes software (Miller et al. 2010) under the GTR + I+G model and run for 10,000,000 generations with a sampling frequency of 100 generations.

**Figure 1.**
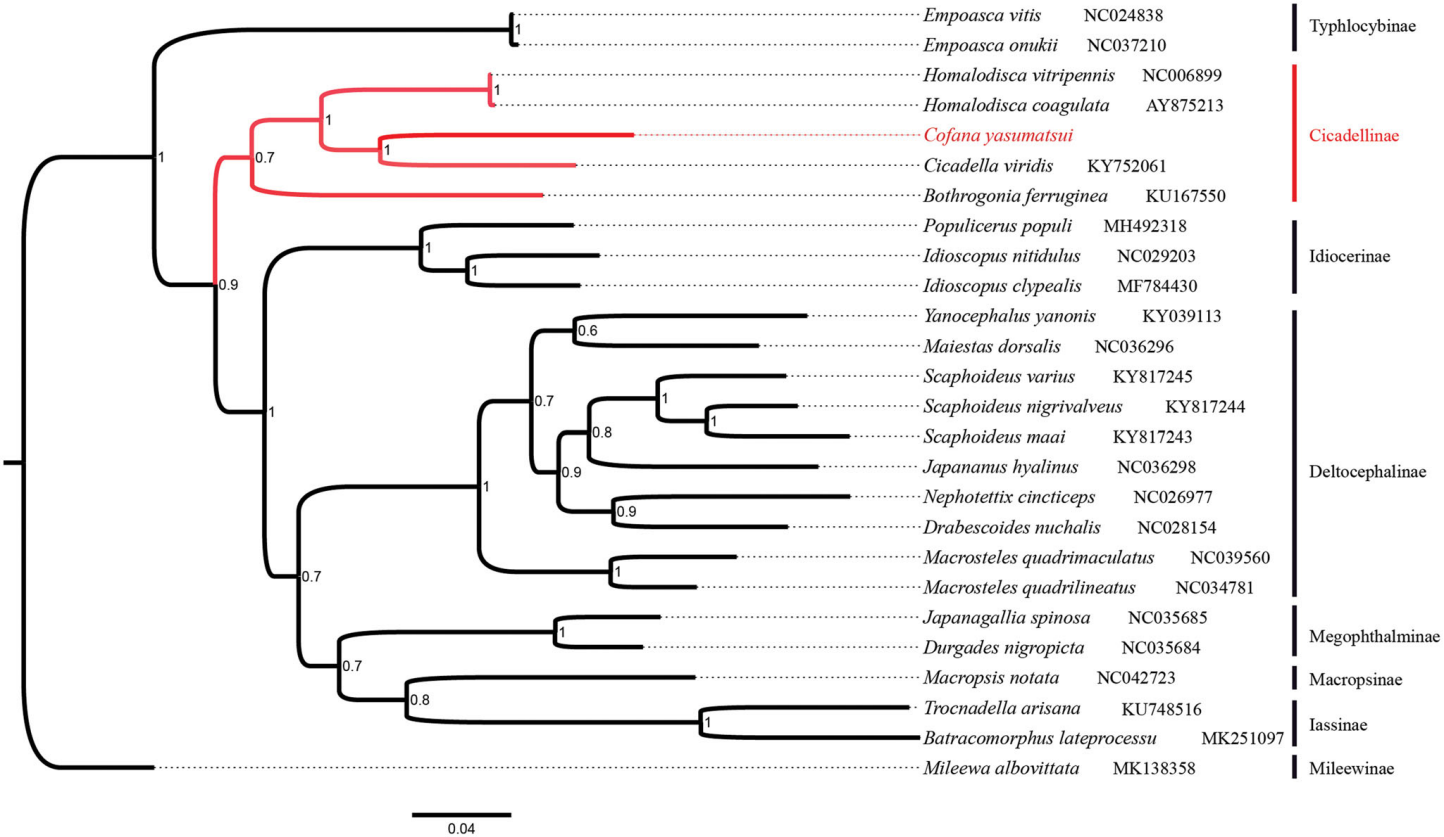
Phylogenetic analyses of *Cofana yasumatsui* based upon the concatenated nucleotide sequences of the 13 PCGs of 26 species. The analysis was performed using MrBayes software. Numbers at nodes are bootstrap values. The accession number for each species is indicated after the scientific name.

The phylogenetic tree showed that *C. yasumatsui* was part of the Cicadellini, which clustered together with *Cicadella viridis*, *Homalodisca coagulata*, *Homalodisca vitripennis,* and *Bothrogonia ferruginea* into a clade, confirming that *C. yasumatsui* is part of the Cicadellinae. The result is consistent with the traditional taxonomy.

## References

[CIT0001] Abascal F, Zardoya R, Telford MJ. 2010. TranslatorX: multiple alignment of nucleotide sequences guided by amino acid translations. Nucleic Acids Res. 38(suppl_2):W7–W13. 20435676 10.1093/nar/gkq291 PMC2896173

[CIT0002] Laslett D, Canbäck B. 2008. ARWEN: a program to detect tRNA genes in metazoan mitochondrial nucleotide sequences. Bioinformatics. 24(2):172–175. 18033792 10.1093/bioinformatics/btm573

[CIT0003] Melichar L. 1926. Monographie der Cicadellinen III. Annales Musei Nationalis Hungarici. 23:273–394.

[CIT0004] Miller MA, Pfeiffer W, Schwartz T. 2010. Creating the CIPRES Science Gateway for inference of large phylogenetic trees. In Proceedings of the Gateway Computing Environments Workshop (GCE), 14 Nov 2010, New Orleans, LA. p. 1–8.

[CIT0005] Yang MF, Meng ZH, Li ZZ. 2017. Hemiptera: Cicadellidae (II): Cicadellinae. Fauna Sinica: Insecta. Vol. 67. Beijing, China: Science Press.

[CIT0006] Young DA. 1979. A review of the leafhopper genus *Cafana* (Homoptera: Cicadellidae). Proceed Entomol Soc Washington. 81(1):1–21.

